# The Political Gender Gap: Gender Bias in Facial Inferences that Predict Voting Behavior

**DOI:** 10.1371/journal.pone.0003666

**Published:** 2008-10-31

**Authors:** Joan Y. Chiao, Nicholas E. Bowman, Harleen Gill

**Affiliations:** 1 Department of Psychology, Northwestern University, Evanston, Illinois, United States of America; 2 Northwestern University Interdepartmental Neuroscience Program, Evanston, Illinois, United States of America; 3 Smith College Neuroscience Program, Northampton, Massachusetts, United States of America; Yale University, United States of America

## Abstract

**Background:**

Throughout human history, a disproportionate degree of political power around the world has been held by men. Even in democracies where the opportunity to serve in top political positions is available to any individual elected by the majority of their constituents, most of the highest political offices are occupied by male leaders. What psychological factors underlie this political gender gap? Contrary to the notion that people use deliberate, rational strategies when deciding whom to vote for in major political elections, research indicates that people use shallow decision heuristics, such as impressions of competence solely from a candidate's facial appearance, when deciding whom to vote for. Because gender has previously been shown to affect a number of inferences made from the face, here we investigated the hypothesis that gender of both voter and candidate affects the kinds of facial impressions that predict voting behavior.

**Methodology/Principal Finding:**

Male and female voters judged a series of male and female political candidates on how competent, dominant, attractive and approachable they seemed based on their facial appearance. Then they saw a series of pairs of political candidates and decided which politician they would vote for in a hypothetical election for President of the United States. Results indicate that both gender of voter and candidate affect the kinds of facial impressions that predict voting behavior. All voters are likely to vote for candidates who appear more competent. However, male candidates that appear more approachable and female candidates who appear more attractive are more likely to win votes. In particular, men are more likely to vote for attractive female candidates whereas women are more likely to vote for approachable male candidates.

**Conclusions/Significance:**

Here we reveal gender biases in the intuitive heuristics that voters use when deciding whom to vote for in major political elections. Our findings underscore the impact of gender and physical appearance on shaping voter decision-making and provide novel insight into the psychological foundations underlying the political gender gap.

## Introduction

*“Cleopatra's nose, had it been shorter, the whole face of the world would have changed.”* - Blaise Pascal

Throughout human history, men have occupied the highest echelons of political power in governments around the world. In recent modern elections, people are more likely than ever before to vote for a woman for highest political offices [1]. Yet from 1960–2002, only 44 women have held their country's highest political office and only 17 of these women served as their nation's President [2]. In 2006, women served as the head of government in only seven countries [3]. If gender bias exists in the electoral process, what factors might underlie this political gender gap in the U.S. and in governments around most of the world?

Contrary to the notion that people use deliberate, rational strategies when deciding whom to vote for in major political elections, research indicates that people use shallow decision heuristics, such as impressions of competence made solely from facial appearance when deciding whom to vote for [4]. For instance, recent evidence has shown that people's impressions of the competence of a political candidate based solely on their facial appearance predict the outcomes of recent U.S. congressional elections [4]. Another recent study showed that differences in facial shape alone between candidates are predictive of who will win or lose an election [5]. Despite the considerable emphasis placed in political elections on educating voters about policy stances that distinguish political candidates and their political parties, voters are as likely to rely on what a candidate looks like as what a candidate stands for when deciding how to cast their votes.

Gender affects how people perceive and evaluate facial appearance [6], [7]. Cultural stereotypes about appropriate social roles for men and women can impact the kinds of facial features that signal attractiveness [6]–[8], dominance [6], [9] and affiliation or approachability [9] in male and female faces. According to social role theory, men are expected by society to be strong and assertive whereas women are expected to be nurturing and sensitive in interpersonal contexts [10], [11]. Consistent with social role theory, male faces are considered more attractive and dominant if they consist of mature facial features (e.g., thick eyebrows, square face, large chins) that are typically associated with physical strength and assertiveness. By contrast, female faces are considered more attractive and affiliative when they consist of immature or ‘baby-faced’ facial features (e.g., thin eyebrows, round face, small chins), which are considered perceptually congruent with the social conception of women as less physically strong and assertive but instead more nurturing and interpersonally sensitive relative to men [6], [12]. Thus, societal expectations about ideal social roles for men and women can influence whom people infer as attractive, dominant and approachable based solely on their facial appearance.

Gender differences in facial appearance also emerge from sexually dimorphic facial features that play an important role in signaling reproductive fitness during mate selection. For instance, facial attractiveness in females has been associated with higher estrogen levels [13] and has been shown to increase during the fertile phase of the menstrual cycle, potentially due to an adaptive mechanism that raises a female's probability of successfully mating when the likelihood of conception is at its highest [14]. Relatedly, a high testosterone-to-estrogen ratio in young men leads to the development of wider cheekbones, mandible and chin, while eyebrows and the central face grow forward and lower facial bones elongate [15]. These kinds of enhanced facial features in male faces are associated with perceived facial dominance in men and actual testosterone levels in young men have been shown to positively correlate with their perceived dominance based solely on facial appearance [16]. Hence, both cultural stereotypes of ideal gender roles and evolutionary processes related to sexual selection affect the perception and evaluation of facial appearance.

In addition to impacting how people evaluate faces, gender affects different facets of leadership, including how people lead and whether or not leaders are perceived as effective. For example, female leaders are more likely to adopt a transformational (e.g., innovative and mentor-like) style of leadership while male leaders are more likely to engage in transactional (e.g., exchange-like) and laissez-faire (e.g., relaxed) styles of leadership [17]. Female and male leaders are perceived as more effective in leadership roles that are congruent with social roles and gender stereotypes [18]. For instance, men are perceived as more effective in more masculine leadership roles (e.g., roles requiring the ability to direct and control people) whereas women are perceived as more effective in feminine leadership roles (e.g., roles requiring interpersonal sensitivity). Similarly, when voters care about policy topics thought to require masculine-style leadership, they prefer male politicians, whereas when they care about topics thought to require feminine-style leadership, voters are more likely to prefer female politicians [19]. Although the influence of gender on facial appearance and leadership effectiveness is widely accepted, whether or not gender impacts the process of leader selection, or how we elect political leaders, remains less well understood.

Given the importance of facial appearance on voter decision-making and the influence of gender on facial appearance and leadership, here we directly tested the hypothesis that gender affects how people judge political candidates based on facial appearance alone as well as the kinds of facial inferences that predict subsequent voting behavior. Participants in the current study judged male and female political candidates on how competent, dominant, attractive and approachable they seemed. Then they saw a series of pairs of political candidates and decided which politician they would vote for in a hypothetical election for President of the United States.

We hypothesized that male politicians would be perceived as more competent and dominant, since these facial attributes are associated with masculinity, whereas female politicians would be perceived as more attractive and approachable, given that these facial attributes are associated with femininity. Moreover, we predicted that both gender of voters and candidates would affect the kinds of facial inferences that predict whom a person votes for.

## Methods

### Participants

Seventy-three university students (38 females, 35 males, *M* in years = 19.52, *SD* = 1.29) participated in the current study. All participants gave written consent prior to participation. The study was approved by the ethics committee at Northwestern University.

### Stimuli

Stimuli consisted of 106 greyscale photographs (46 females, 60 males) of congressional candidates from the 2006 House of Representative election. All photos were first obtained from the Cable News Network (CNN) website and then standardized for background color, size and luminosity. Only two-candidate races with 1 Republican and 1 Democrat were included in order to control for potential effects of third party candidates. Any congressional candidate that was considered potentially familiar due to a high-profile leadership position within the House of Representatives (e.g., Nancy Pelosi) was not included as stimuli.

### Task Procedure

**Figure 1:**
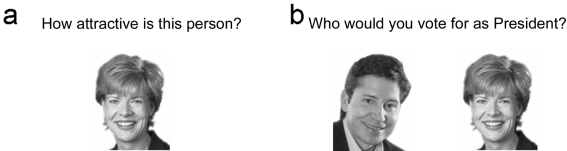
Example of two tasks in the current experiment. a) In the facial judgment task, participants indicated how competent, attractive, approachable and dominant each candidate appeared on a seven-point Likert scale (1 = not at all, 7 = very much). b) In the hypothetical voting task, participants indicated which of the two candidates they would vote for in a U.S. Presidential election, considering all other factors equivalent.

Participants completed two behavioral tasks: a facial judgment task (see Figure 1a) and a hypothetical U.S. Presidential election voting task (see Figure 1b). First, participants saw a face of a political candidate for 1 second and then judged how competent, dominant, approachable and attractive the face seemed on a 7-point Likert scale (1 = not at all to 7 = very much). Second, participants saw a series of pairs of political candidates from the 2006 House of Representatives election and were asked to decide which of the two candidates they would vote for in a U.S. Presidential election, imagining that all other attributes of the candidates were equal. Order of presentation of faces was randomized and the location (e.g., right or left side of the screen) of winners and losers as defined by the 2006 HR election was counterbalanced across participants.

We controlled for participants' familiarity with candidates in the following ways. First, as stated earlier, any congressional candidate that was considered potentially familiar due to a high-profile leadership position within the House of Representatives (e.g., Nancy Pelosi) was not included as stimuli. Second, after the two experimental tasks, we measured participants' perceived familiarity with candidates by showing them each candidate one at a time asking them to indicate whether or not they recognized the candidate from any prior election. Results from this familiarity test indicated that participants recognized less than 10% of all candidates (*M* = 8.9%, *SD* = 19.8%). Third, in the exit survey, participants were asked if they had ever voted in an election and whether or not they found anything strange about the experiment. Results from this exit survey indicated that only 17% of participants had participated in an election before and no participant indicated that they recognized any political candidate in exit survey responses. Taken together, these results suggest that familiarity with candidates was negligible across participants in the current experiment.

### Data Analyses

Data analyses consisted of four parts. First, to determine the effect of gender of candidate and gender of voter on inferences from facial appearance, four separate 2 (gender of candidate)×2 (gender of participant) repeated-measures ANOVAs were conducted on ratings of the competence, dominance, attractiveness and approachability of political candidates. Second, to determine the relationship between gender, facial inferences and simulated voting behavior, standard multiple regression analyses were conducted between percentage of participants who voted for a candidate as the dependent variable and facial inferences of competence, dominance, attractiveness and approachability, controlling for incumbency and party of the candidate, as independent variables, and candidate as the unit of analysis. Third, to determine the relationship between gender, facial inferences and actual election outcomes in the 2006 House of Representatives election, standard multiple regression analyses were conducted between percentage of participants who voted for a candidate as the dependent variable and facial inferences of competence, dominance, attractiveness and approachability, controlling for incumbency and party of the candidate, as independent variables, and candidate as the unit of analysis. Fourth, to assess the relationship between the margin of simulated victory and margin of actual electoral victory, we conducted a bivariate correlation analysis across all candidates between percentage of actual votes received in the 2006 House of Representatives election as and percentage of votes received in the simulated Presidential election conducted in the current experiment.

## Results

### Gender and facial appearance

#### Competence

There was a significant main effect of gender of candidate on impressions of competence, *F* (1, 71) = 4.37, *p*<0.0001 (see Table 1). All voters perceived male politicians as significantly more competent compared to female politicians, *t* (73) = 2.11, *p*<0.05. There was no significant interaction between gender of candidate and gender of participant or significant main effect of gender of participant on facial appearance of competence (all *p*'s>0.05).

**Table 1:** Results from facial judgment task as a function of gender of candidates and voters (in M±SD).

	Female political candidates	Male political candidates	Difference score	*P* value
*All voters (n = 73)*				
Competent	4.50 (1.15)	4.70 (0.96)	−0.21	*<0.05*
Dominant	4.17 (0.84)	4.43 (0.74)	−0.26	*<0.0001*
Attractive	3.60 (0.89)	3.11 (0.95)	0.49	*<0.0001*
Approachable	4.83 (0.77)	4.41 (0.78)	0.43	*<0.0001*
*Female voters (n = 38)*				
Competent	4.48 (1.38)	4.73 (1.09)	−0.25	ns
Dominant	3.98 (0.71)	4.40 (0.75)	−0.42	*<0.0001*
Attractive	3.64 (0.91)	3.22 (1.01)	0.43	*<0.01*
Approachable	4.66 (0.77)	4.17 (0.64)	0.63	*<0.002*
*Male voters (n = 35)*				
Competent	4.51 (0.86)	4.67 (0.81)	−0.16	*<0.05*
Dominant	4.37 (0.93)	4.48 (0.73)	−0.11	ns
Attractive	3.57 (0.87)	3.00 (0.88)	0.56	*<0.0001*
Approachable	5.02 (0.74)	4.66 (0.88)	0.86	*<0.0001*

#### Dominance

There was a significant main effect of gender of candidate on impressions of dominance, *F* (1, 71) = 14.25, *p*<0.0001 (see Table 1). All voters perceived male politicians as significantly more dominant compared to female politicians, *t* (72) = 3.70, *p*<0.0001. There also was a significant interaction between gender of candidate and gender of voter, *F* (1, 71) = 5.09, *p*<0.03, on perceived dominance. Male voters did not perceive a significant difference in how dominant male and female candidates appeared to be, whereas for female voters, male political candidates were perceived as significantly more dominant compared to female political candidates, *t* (34) = 5.03, *p*<0.0001.

There was no significant main effect of gender of voter on perception of dominance (*p*>0.05).

#### Attractiveness

There was a significant main effect of gender of candidate on impressions of attractiveness, *F* (1, 71) = 53.64, *p*<0.0001 (see Table 1). Across all voters, female politicians were perceived as significantly more attractive relative to male politicians, *t* (72) = 7.31, *p*<0.0001. There was no significant main effect of gender of participant on attractiveness or significant interaction between gender of candidate and gender of participant (all *p*'s>0.05).

#### Approachability

There was a significant main effect of gender of candidate on impressions of approachability, *F* (1, 71) = 44.63, *p*<0.0001 (see Table 1). Female politicians were perceived as significantly more approachable relative to male politicians, *t* (72) = 6.70, *p*<0.0001. There was also a significant main effect of gender of voter on impressions of approachability, *F* (1, 71) = 6.66, *p*<0.01. Male voters rated all politicians as more approachable relative to female voters, *t* (71) = 2.58, *p*<0.01. There was no significant interaction between gender of participant and gender of candidate.

### Relationship between gender, facial appearance and simulated voting for President

Across all voters and candidates, competence was a significant predictor of simulated voting behavior [*r* (106) = 0.64, *p*<0.0001] (see Table 2). Importantly, gender of political candidate affected types of facial judgments that significantly predicted hypothetical voting for male and female Presidential candidates. People were significantly more likely to vote for more competent looking male candidates [*r* (60) = 0.59, *p*<0.003], and female candidates [*r* (46) = 0.76, *p*<0.01]. Intriguingly though, male candidates were also more likely to win votes if they appeared approachable [*r* (60) = 0.55, *p*<0.009], while female candidates were more likely to win votes if they were more attractive [*r* (46) = 0.75, *p*<0.001].

**Table 2:** Results from multiple linear regression model with facial inferences as predictor variables and percentage of votes won in simulated U.S. Presidential voting as the criterion variable, controlling for political incumbency and party.

Predictor	All candidates (n = 106)	Female candidates (n = 46)	Male candidates (n = 60)
*All voters*			
Competence	0.43***	0.41*	0.41*
Dominance	0.09	−0.01	0.06
Attractiveness	0.19	0.54**	−0.01
Approachability	0.17	−0.09	0.34*
Accounted variance (*R* ^2^)	53.4%	70.6%	48.7%
*Female voters*			
Competence	0.43***	0.58*	0.37*
Dominance	0.09	−0.16	0.13
Attractiveness	0.13	0.42*	−0.05
Approachability	0.20*	−0.08	0.35*
Accounted variance (*R^2^*)	51.7%	63.6%	49.3%
*Male voters*			
Competence	0.36***	0.31*	0.38*
Dominance	0.12	0.04	0.00
Attractiveness	0.28*	0.61***	0.16
Approachability	0.13	−0.07	0.24
Accounted variance (*R^2^*)	50.0%	70.8%	41.7%

Each candidate is the unit of analysis (Standardized Beta Coefficients * *<0.05*; ** *0.001*; *** *0.0001*).

**Figure 2:**
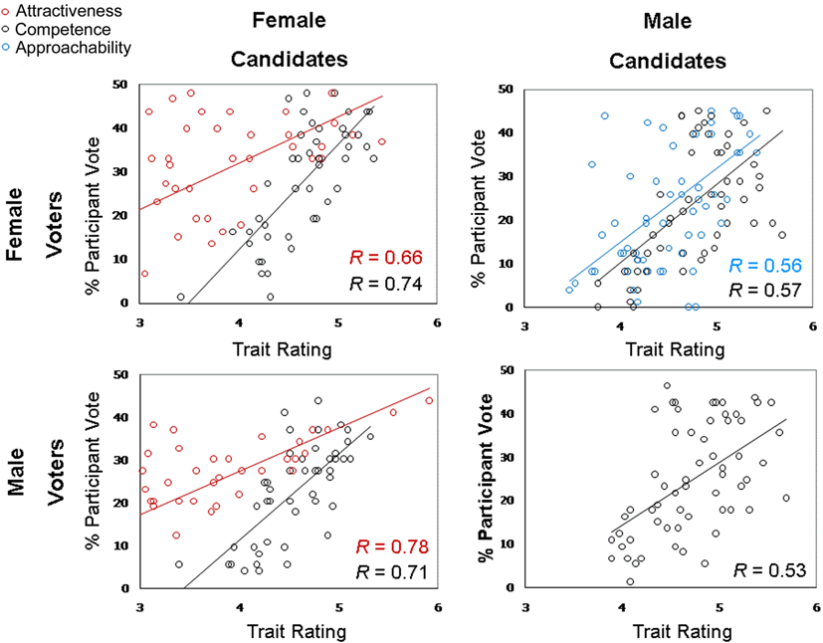
Scatterplots of percentage of female (top row) and male (bottom row) voters in the current experiment who voted for female (left column) and male (right column) political candidates as a function of inferred competence (black circles), approachability (blue circles) and attractiveness (red circles). Each point represents a congressional candidate in the 2006 House of Representative election.

Gender of voter also affected the types of facial inferences that predicted voting preferences (see Table 2). Candidates that appeared more competent were more likely to win votes of male [*r* (106) = 0.60, *p*<0.0001] (see Figure 2, bottom row) and female [*r* (106) = 0.61, *p*<0.0001] voters (see Figure 2, top row). In addition, male voters were significantly more likely to vote for candidates that appeared attractive [*r* (106) = 0.56, *p*<0.007] (see Figure 2, bottom left), while female voters were significantly more likely to vote for candidates that seemed approachable [*r* (106) = 0.46, *p*<0.03] (see Figure 2, top right).

Taking both gender of the voter and gender of candidate into account again yielded divergent types of facial inferences that predicted voting (see Table 2). Female voters were more likely to vote for male candidates who appeared both competent [*r* (60) = 0.57, *p*<0.004] (see Figure 2, top right) and approachable [*r* (60) = 0.56, *p*<0.007] (see Figure 2, top right), but for female candidates who appeared both competent [*r* (46) = 0.74, *p*<0.002] (see Figure 2, top left) and attractive [*r* (46) = 0.66, *p*<0.02] (see Figure 2, top left). Critically, male voters were more likely to vote for male candidates only if they appeared competent [*r* (60) = 0.53, *p*<0.005] (see Figure 2, bottom right), whereas they were more likely to vote for female candidates if they appeared both attractive [*r* (46) = 0.78, *p*<0.0003] (see Figure 2, bottom left) and competent [*r* (46) = 0.72, *p*<0.03] (see Figure 2, bottom left).

### Relationship between gender, facial appearance and actual Congressional election outcomes

Although we used a simulated voting task (e.g., Who would you vote for as U.S. President?) that was distinct from the actual political election (e.g., U.S. House of Representatives) all of the candidates ran in, we examined the relationship between facial inferences and actual Congressional election outcomes.

Across all voters and candidates, perceived competence (*p*<0.05, see Table 3) and dominance (*p*<0.05, see Table 3) were significant predictors of actual election outcomes. Gender of political candidate affected types of facial judgments that significantly predicted actual election outcomes for male and female House of Representative candidates. In particular, perceived competence significantly predicted actual election outcomes for male candidates (*p*<0.05, see Table 3), but not female candidates.

**Table 3:** Results from multiple linear regression model with facial inferences as predictor variables and percentage of votes won in the 2006 U.S. House of Representatives election as the criterion variable, controlling for political incumbency and party.

Predictor	All candidates (n = 106)	Female candidates (n = 46)	Male candidates (n = 60)
*All voters*			
Competence	−0.20*	−0.09	−0.26*
Dominance	0.20*	0.15	0.20
Attractiveness	0.07	0.09	0.03
Approachability	0.04	0.03	0.11
Accounted variance (*R* ^2^)	65.0%	78.6%	58.7%
*Female voters*			
Competence	−0.08	−0.01	−0.16
Dominance	0.11	0.04	0.11
Attractiveness	−0.01	0.09	−0.02
Approachability	0.08	0.04	0.18
Accounted variance (*R^2^*)	63.4%	77.6%	57.7%
*Male voters*			
Competence	−0.20*	−0.07	−0.25*
Dominance	0.15	0.16	0.12
Attractiveness	0.18*	0.10	0.17
Approachability	−0.04	0.01	−0.04
Accounted variance (*R* ^2^)	66.3%	79.6%	59.5%

Each candidate is the unit of analysis (Standardized Beta Coefficients * *<0.05*; ** *0.001*; *** *0.0001*).

Gender of voter also affected the types of facial inferences that predicted actual election outcomes from the 2006 House of Representatives race. Perceived competence by male voters, but not female voters significantly predicted actual election outcomes (*p*<0.05, see Table 3).

Taking both gender of voter and gender of candidate into account revealed divergent kinds of facial inferences that predicted actual election outcomes. Perceived competence of male candidates by male voters (*p*<0.05, see Table 3) predicted actual election outcomes for male Congressional candidates. However, facial inferences by male and female voters did not significantly predict actual election outcomes for female Congressional candidates.

### Relationship between simulated Presidential voting and actual Congressional election outcomes

Across all candidates, simulated Presidential voting in the current experiment significantly and positively correlated with actual election outcomes from the 2006 House of Representative race [*r* (106) = 0.24, *p*<0.01].

## Discussion

Our results reveal a gender bias in the intuitive heuristics voters use when evaluating political candidates and deciding who to vote for. Voters perceive the faces of male politicians as more competent and dominant relative to female politicians whereas female politicians are perceived as more attractive and approachable relative to male politicians. Given the known importance of facial inferences of competence in predicting actual electoral outcomes [4], this finding suggests that one factor underlying the political gender gap is the impression that voters have of male politicians as more competent than female politicians.

Why do the faces of male politicians signal greater competence and dominance to voters relative to the faces of female politicians who appear more attractive and affiliative? One possible explanation posited by thin slice theory [20] is that voters are able to accurately infer the actual competence of politicians solely from facial appearance and thus, in this study, voters accurately glean from facial appearance that male politicians really are more competent relative to female politicians. However, this explanation is unlikely for two reasons. First, meta-analytic evidence indicates that female and male leaders do not differ in actual effectiveness or competence across a range of leadership roles (e.g., managers to CEOs), irrespective of their preferred leadership style (e.g., transformational vs. transactional) [18]. Second, empirical work examining the effectiveness of governments led by women has shown that female politicians outperform male politicians in several ways [21], [22]. For instance, female politicians in India are less likely to be corrupt and more likely to provide public goods in a fair and affordable manner relative to their male counterparts [21]. Although the effectiveness of male and female politicians is difficult to wholly examine due to the persistent lack of representation of female politicians in the highest echelons of modern government, a growing body of evidence indicates that male and female politicians do not differ in actual leadership effectiveness.

An alternative explanation based on social role theory is that voters construe positions of top political leadership, such as the President of the United States, as inherently more masculine in nature (e.g., requiring the ability to direct and control others) and thus, perceive faces of male politicians, which contain more masculine facial features, as more competent or effective in that political role relative to faces of female politicians, which contain more feminine facial attributes. Conversely, because faces that contain feminine or baby-faced facial features are perceived as more attractive and affiliative, voters perceive female politicians as significantly more attractive and affiliative relative to male politicians. Given the evidence showing that men and women do not differ in leadership effectiveness, we suggest that voters' perception of male politicians as more competent relative to female politicians observed in the current study are more likely driven by cultural stereotypes of who is more likely to be competent rather than accurate assessments of who is actually competent.

The present findings further indicate that gender stereotypes predispose us to value divergent qualities in leaders, such as attractiveness in female politicians and approachability in male politicians, when deciding whom to vote for in major elections. Although impressions of competence from facial appearance are ubiquitously predictive of voting behavior, both male and female voters are more likely to vote for female politicians who not only appear competent, but also attractive. Moreover, female voters are more likely to vote for male politicians who not only appear competent, but also approachable. These results corroborate a growing body of research demonstrate the potency of facial appearance in political decision-making [4], [5] and highlight the gender bias in intuitive heuristics used by voters when evaluating the faces of male and female political candidates and deciding who to vote for.

Why does facial attractiveness matter to the electoral success of female but not male politicians? One possible explanation is that the current findings are simply a result of experimental task demand. That is, because voters judged female politicians as more attractive relative to male politicians prior to the voting phase of the experiment, they valued attractiveness in female politicians to a greater extent relative to male politicians and were more likely to vote for attractive female politicians relative to attractive male politicians. However, this explanation is not likely for two reasons. If voters' facial judgments of attractiveness influenced their voting behavior for female and male politicians, then their voting behavior should have been similarly affected by the other kinds of facial judgments, including competence, dominance and approachability made prior to the voting phase of the current study. As discussed earlier, all voters judged male politicians as appearing more competent relative to female politicians; yet, competence was equally predictive of voting behavior for both male and female political candidates. Similarly, all voters judged male politicians as appearing more dominant relative to female politicians; however, perceived facial dominance was not predictive of voting behavior for either female or male political candidates. Hence, it is not likely that our findings are solely a function of task demand.

Another possible explanation based on the ‘halo effect’ is the notion that voters perceive attractive female politicians as good at leadership because of a cognitive bias to unconsciously associate attractiveness with superior ability in other, unrelated personality dimensions, such as intelligence, talent, kindness and honesty [23]. However, the ‘halo effect’ cannot explain why in the current study facial attractiveness was associated with voting for female but not male politicians.

A third, more nuanced, explanation of the current findings based on evolutionary theory is that people automatically evaluate faces using a core constellation of intuitive heuristics critical for other kinds of adaptive decision-making, such as mate selection. Akin to leadership selection, men and women value different qualities in heterosexual mate selection. Across cultures, men are more likely to prefer women who are physically attractive, whereas women are more like to prefer men who have high social status or demonstrate the ability to garner resources [24]. We suggest that both male and female voters value physical attractiveness in female but not male politicians because this adaptive quality is emphasized in mate selection and thus engenders a broader cultural expectation that attractive women are more deserving of high social status roles not only in the domain of sexual selection, but also leadership selection. Similarly, female voters value not only competence but also approachability in male politicians due to the importance of qualities such as kindness and warmth in female selection of male long-term partners [25].

Our findings also indicate that voters in the current study showed similar, though not identical, preferences to people who voted in the actual 2006 House of Representatives election. Margin of victory for politicians in the hypothetical Presidential election was associated with margin of victory in the actual 2006 House of Representatives election, suggesting that voter preferences observed here generalize to preferences of the general electorate across different kinds of political offices. Additionally, candidates who were perceived as more attractive by men were more likely to win votes in the actual Congressional election. However, there were notable distinctions in the relationship between gender, facial inference and voting behavior in current simulated Presidential election outcomes versus actual Congressional election outcomes. First, candidates who were perceived as competent were less likely to win the actual Congressional election, whereas candidates who appeared dominant were more likely to win votes in the actual Congressional election. Second, none of the facial inferences that predicted simulated voting for female Presidential candidates were predictive of their actual Congressional outcomes. One possible explanation for this discrepancy between the intuitive heuristics that predict simulated Presidential voting in the current experiment and actual Congressional outcomes is that there is a fundamental difference in voter preferences in the laboratory settings relative to in real elections. However, this explanation is not likely due to prior research showing that facial inferences made laboratory settings predict actual election outcomes [4], [5].

Another possibility is that the intuitive heuristics used by voters during leader selection varies as a function of the prestige and selectivity of the political office. We suggest that this alternative explanation is more likely given that the degree of selectivity required in choosing a leader for the highest political office (e.g., one leader instead of one out of several leaders at the same rank) is more analogous to the degree of selectivity required when choosing a mate and thus, gender biases evident in mate selection may exert greater influence in leader selection, particularly under these circumstances. For instance, while political gender gaps exist throughout the political ladder, they are most visible in the very highest echelons of governments around the world. Over 40 women ran for office in the 2006 U.S. House of Representatives election alone, whereas not once has there been a female major party candidate for President in U.S. history.

In sum, here we identify two psychological attributes of the voter that likely contribute to the political gender gap. First, gender stereotypes may bias voters to value male politicians over female politicians simply because they possess facial features that signal qualities associated with effective leaders. Second, endowed with intuitive heuristics for selecting optimal mates, voters may unconsciously apply this set of core heuristics when making other kinds of seemingly unrelated, but important, social decisions, such as deciding whom to vote for. While the ideal personal characteristics of a good political leader at first glance appear largely distinct from those that comprise a good mate, cognitive remnants of our evolutionary history may predispose us with similar gender biases, which are incongruent with modern cultural ideals of gender equality in political representation and political power.

Notably, exposure to female politicians has been shown to reduce use of gender stereotypes when evaluating leadership effectiveness as well as overall negative biases towards female leaders [20]. While the current findings demonstrate gender biases in facial inferences that affect voting behavior, as women become an increasingly visible presence in electoral politics and government, voters may learn to reduce their reliance on cognitive shortcuts, such as gender stereotypes and intuitive heuristics [10].
